# Dietary Rumen-Protected Gamma-Aminobutyric Acid Supplementation to Alleviate Stress in Beef Cattle Caused by Long-Distance Transport by Improving Antioxidant Capacity, Immune Function, and Hormonal Balance: A Related Study

**DOI:** 10.3390/ani16182839

**Published:** 2026-09-09

**Authors:** Tao Chen, Meijuan Bai, Lizhi Wang

**Affiliations:** 1 Institute of Animal Nutrition, Sichuan Agricultural University, Chengdu 611130, China; chentao@stu.sicau.edu.cn; 2 Hunan Perfect Biotechnology Co., Ltd., Changsha 410152, China; 13618460806@163.com

**Keywords:** transport stress, γ-aminobutyric acid, beef cattle, antioxidant capacity, hormone levels

## Abstract

γ-aminobutyric acid (GABA), an inhibitory neurotransmitter, plays an important role in regulating stress responses through modulation of the hypothalamic–pituitary–adrenal axis, neuroendocrine activity, antioxidant defense, and immune function. Therefore, this study evaluated the effects of dietary rumen-protected γ-aminobutyric acid (RP-GABA) supplementation on alleviating long-distance transport-induced stress in beef cattle. Forty-eight healthy Simmental bulls were randomly assigned to four treatment groups: CON, G-L, G-M, and G-H, receiving RP-GABA at 0, 300, 500, and 700 mg/kg of concentrate dry matter, respectively. The experiment lasted 53 days, including a 21-day pre-transport feeding period, a 2-day transportation period, and a 30-day post-transport feeding period. Samples were collected on experimental Days 21, 23, and 54. The results showed that long-distance transport impaired antioxidant status, immune function, and stress hormone secretion in beef cattle. The responses to different dietary RP-GABA supplementation levels varied among serum parameters. Long-distance transport reduced triiodothyronine and thyroxine (T4) concentrations, while the G-L group alleviated these reductions and maintained higher levels than the CON group after transport (Day 23). The G-M group enhanced glutathione peroxidase (GSH-Px) activity after transport (Day 23) (*p* < 0.001) and promoted the elevation of interleukin (IL)-4 levels both before and after long-distance transport (Days 21 and 23). The G-H group significantly decreased serum cortisol concentration after transport (Day 23), while reducing epinephrine levels and increasing glutathione peroxidase activity (all *p* < 0.001). Moreover, the G-H group further enhanced the increases in IL-4 and total antioxidant capacity before and after transport (Days 21 and 23), and increased IL-6 concentrations before and after transport (Days 21 and 54). Regarding dose-specific effects, the G-L group showed limited effects on stress-related hormones, immune function, and antioxidant capacity, whereas the G-H group enhanced some antioxidant and immune-related responses but had higher pre-transport stress hormone concentrations. In contrast, the G-M group exhibited a relatively balanced response across stress hormone levels, antioxidant status, and immune regulation, characterized by higher T4 (*p* = 0.003), GSH-Px (*p* = 0.001), and IL-4 (*p* = 0.006) concentrations and lower alkaline phosphatase (*p* = 0.005) and tumor necrosis factor-alpha concentrations. Therefore, under the conditions of this study, dietary supplementation with 500 mg/kg RP-GABA in concentrate may represent a potential level for maintaining balanced physiological responses in beef cattle.

## Simple Summary

Long-distance transport is a critical component of modern beef cattle production systems, particularly in China, but it can adversely affect immune function, antioxidant status, and gastrointestinal health. γ-aminobutyric acid (GABA), an inhibitory neurotransmitter, has been reported to regulate stress responses through modulation of stress hormone secretion, antioxidant capacity, and immune function, suggesting its potential to alleviate transport-induced stress in cattle. This study evaluated the effects of dietary supplementation with different levels of rumen-protected γ-aminobutyric acid (RP-GABA) on production performance, serum biochemical parameters, stress hormone secretion, immunity, and antioxidant capacity in beef cattle subjected to long-distance transport. The results showed that RP-GABA supplementation modulated stress-related hormonal responses and improved antioxidant and immune status, with effects varying among supplementation levels. These findings provide insights into the potential application of RP-GABA as a nutritional strategy to enhance stress resilience in beef cattle during long-distance transport.

## 1. Introduction

Long-distance transport is an important component of modern beef cattle production systems. During transport, cattle are exposed to multiple stressors, including overcrowding, physical jostling, limited access to feed and water, and environmental changes. These stressors can activate the hypothalamic–pituitary–adrenal (HPA) axis and the sympathetic nervous system, thereby inducing a series of physiological stress responses in beef cattle [1,2,3]. The stress response is characterized by alterations in biochemical parameters, including increased serum cortisol (COR), catecholamine, and acute-phase protein concentrations, as well as metabolic disturbances such as negative energy balance, muscle glycogen depletion, and enhanced lipolysis [4,5,6]. Previous studies have demonstrated that transport stress exerts adverse effects on immune function, antioxidant status, and respiratory and gastrointestinal health in beef cattle, ultimately impairing growth performance and feed efficiency and resulting in economic losses to the beef cattle industry [7,8,9].

γ-aminobutyric acid (GABA) is the major inhibitory neurotransmitter in the central nervous system of animals and plays important roles in regulating neuronal excitability, feed intake, energy metabolism, endocrine function, and immune responses [10,11]. Previous studies have shown that GABA exhibits stress-alleviating effects by improving nutrient digestibility and growth performance in heat-stressed beef cattle, enhancing immune function and antioxidant capacity in heat-stressed dairy cattle, and improving rumen fermentation and growth development in calves subjected to weaning stress [12,13,14]. These findings suggest that RP-GABA may have the potential to attenuate transport-associated stress through neuroendocrine, antioxidant, and immune regulatory pathways. However, whether RP-GABA can alleviate transport stress in beef cattle remains largely unexplored. Conventional GABA is easily degraded by rumen microorganisms in ruminants, which reduces its bioavailability; in contrast, rumen-protected GABA (RP-GABA) can bypass ruminal degradation, be absorbed in the small intestine, and exert biological functions in the body [15]. Previous studies reported that GABA supplementation at 1.2 g/day alleviated transport stress in animals, while 3 g/day GABA supplementation alleviated heat stress in beef cattle [16,17]. Based on previous studies, dose conversion of reported GABA supplementation levels, and the characteristics of RP-GABA, three supplementation levels of 300, 500, and 700 mg/kg of concentrate dry matter (DM) were selected for the present study. The objective of this study was to evaluate the effects of different dietary RP-GABA supplementation levels on production performance, serum biochemical parameters, hormone levels, immune function, and antioxidant capacity in beef cattle during long-distance transport, and to identify an appropriate supplementation level for alleviating transport stress. The results of this study may provide a scientific basis for the application of RP-GABA in mitigating transport stress in beef cattle.

## 2. Materials and Methods

### 2.1. Ethical Statement

All experimental procedures were approved by the Animal Ethics Committee of Sichuan Agricultural University and conducted in accordance with current animal welfare guidelines (Approval No. SCAUAC201408-3).

### 2.2. Experimental Animals and Design

Forty-eight healthy Simmental bulls with an average initial body weight of 181.95 ± 2.92 kg were selected for this study. A completely randomized design was implemented, and bulls were randomly allocated into four groups (*n* = 12 per group): CON, G-L, G-M, and G-H. RP-GABA was supplemented at 0, 300, 500, and 700 mg/kg of concentrate DM, respectively. A stepwise dilution premixing method was used to ensure homogeneous distribution of RP-GABA in the concentrate (pelleted feed). Briefly, 300, 500, or 700 g of RP-GABA was thoroughly mixed with corn to prepare a 20 kg preliminary premix. The 20 kg preliminary premix was then thoroughly mixed with an additional 80 kg of corn to obtain a 100 kg secondary premix, which was subsequently mixed thoroughly with 900 kg of the basal diet to obtain the final experimental diet. During the experimental period, each bull was offered the experimental concentrate twice daily (1 kg per feeding, 2 kg/day in total), which was thoroughly mixed with hay. Cattle were fed individually, and strict feed bunk management was implemented to monitor concentrate consumption. Residual concentrate was maintained at a low level in all groups (orts < 5% of offered), minimizing variation in actual RP-GABA intake among animals. RP-GABA was purchased from Hunan Perfect Biotechnology Co., Ltd. (Changsha, China) and contained ≥50% GABA, ≥0.5% taurine, hydrogenated fatty acids, and other excipients. The composition and nutrient levels of the basal diet are presented in Table 1.

The experiment lasted 53 days, consisting of a 21-day pre-transport feeding period, a 2-day transport period, and a 30-day post-transport feeding period. During the pre-transport period (Days 1–21), cattle were fed in Si’ping City, Jilin Province, China, where the ambient temperature ranged from 15 to 22 °C throughout the pre-transport feeding period. On Day 22, cattle were loaded and transported by road to Ba’zhong City, Sichuan Province, China, using a 9.6-m double-deck heavy-duty truck. The stocking density during transport was approximately 0.9–1.0 m^2^/head, with a duration of approximately 28 h and a total distance of approximately 2800 km. Based on the practical conditions of long-distance cattle transportation in China, as well as to avoid injuries caused by animal trampling or slipping due to feeding and watering during transit, feed and water deprivation was applied during transport to simulate commercial transportation conditions and induce a representative transport stress response. Upon arrival on Day 23, cattle were immediately provided with feed and water and entered a 30-day post-transport recovery feeding period (Days 24–53). During this period, the ambient temperature ranged from 20 to 28 °C. Body weight was recorded on Days 1 and 54, and physiological measurements and blood samples were collected on Days 21 (before transport), 23 (immediately after transport), and 54 (after the post-transport feeding period).

### 2.3. Production Performance

The experimental cattle were deprived of feed and water for 12 h before weighing on Days 1 and 54. Body weight was recorded on the following morning before feeding. The initial body weight (IBW) and final body weight (FBW) were recorded on Days 1 and 54, respectively, and average daily gain (ADG) was calculated based on the IBW and FBW values.

### 2.4. Breathing Rate and Temperature

Breathing rate was measured by manual counting on Days 21, 23, and 54. The measurement method was as follows: when the beef cattle were at rest, a stopwatch was used to continuously record the number of lateral abdominal movements (or nostril flaring) for 1 min. The measurement was performed three times, and the average value was calculated as the final breathing rate for each measurement period [18]. Meanwhile, an electronic thermometer (DIGI-Vet SC 12, KRUUSE, Langeskov, Denmark) was inserted approximately 3–5 cm into the rectum to measure rectal temperature [19].

### 2.5. Collection of Blood Samples

Blood samples were collected from the jugular vein of each animal at 8:00 a.m. on Days 21, 23, and 54. Sampling on Day 21 represented the physiological status before transportation after the pre-transport supplementation period; Day 23 reflected the acute response to long-distance transport stress; and Day 54 represented the recovery status after the post-transport feeding period. Before sampling on Days 21 and 54, feed and water were withheld for 12 h, whereas samples collected on Day 23 were obtained immediately upon arrival at the destination after feed and water deprivation during the transport period. Four tubes of blood were collected from each animal at each sampling time point. After standing for at least 30 min, the blood samples were centrifuged at 3000 r/min for 10 min at 4 °C to separate the serum. After centrifugation of the four blood tubes, the obtained serum samples were transferred into Eppendorf tubes and stored at −20 °C until analysis of serum biochemical parameters, hormone levels, immune parameters, and antioxidant parameters, respectively.

### 2.6. Laboratory Analysis

Serum biochemical parameters, including alanine transaminase (ALT), aspartate transaminase (AST), alkaline phosphatase (ALP), lactate dehydrogenase (LDH), creatine kinase (CK), total protein (TP), albumin (ALB), urea (UREA), creatinine (CREA), glucose (GLU), total cholesterol (TC), triglycerides (TG), high-density lipoprotein cholesterol (HDL-C), low-density lipoprotein cholesterol (LDL-C), lipopolysaccharide (LPS), and histamine (HIS), were determined using an automatic biochemical analyzer (Hitachi 3100, Hitachi Ltd., Tokyo, Japan).

Serum malondialdehyde (MDA), total antioxidant capacity (T-AOC), glutathione peroxidase (GSH-Px), superoxide dismutase (SOD), and catalase (CAT) activities were determined using commercial kits provided by the Nanjing Jiancheng Biotechnology Research Institute Co., Ltd. (Nanjing, China). The corresponding catalog numbers and intra-assay/inter-assay coefficients of variation (CV/CV_i_) for each parameter were as follows: CAT (A007-1-1, CV = 1.7%), GSH-Px (A005-1-2, CV = 3.56%, CV_i_ = 6.8%), MDA (A003-1-2, CV = 2.3%, CV_i_ = 5.34%), T-AOC (A015-2-1, CV = 3.6%, CV_i_ = 6.8%), and SOD (A001-3, CV = 1.2%).

Serum concentrations of interleukin (IL)-1β, IL-4, IL-6, IL-10, and tumor necrosis factor-alpha (TNF-α) were determined using commercial assay kits provided by Beijing Solarbio Science & Technology Co., Ltd. (Beijing, China). The corresponding catalog numbers and CV/CV_i_ for each parameter were as follows: IL-1β (SEKB-0363, CV < 10%, CV_i_ < 10%), IL-4 (SEKB-0364, CV < 10%, CV_i_ < 10%), IL-6 (SEKB-0365, CV < 10%, CV_i_ < 10%), IL-10 (SEKB-0362, CV < 10%, CV_i_ < 10%), and TNF-α (SEKB-0303, CV < 10%, CV_i_ < 10%).

Serum COR, adrenocorticotropic hormone (ACTH), triiodothyronine (T3), thyroxine (T4), and epinephrine (EPI) concentrations were determined using commercial assay kits provided by Shanghai Enzyme-linked Biotechnology Co., Ltd. (Shanghai, China). The corresponding catalog numbers and CV/CV_i_ for each parameter were as follows: COR (YJ32911, CV < 10%, CV_i_ < 15%); ACTH (YJ33028, CV < 10%, CV_i_ < 15%); T3 (YJ34785H, CV < 10%, CV_i_ < 15%); T4 (YJ32956, CV < 10%, CV_i_ < 15%); and EPI (YJ32879, CV < 10%, CV_i_ < 15%).

### 2.7. Statistical Analysis

Statistical analyses were performed using SPSS software (v27.0 for Windows; SPSS Inc., Chicago, IL, USA). Growth performance parameters were analyzed using one-way analysis of variance (ANOVA), followed by LSD pairwise comparisons to explore differences among treatment groups. For repeated measurements, the linear mixed model (MIXED) procedure was used, with dietary RP-GABA supplementation, time, and their interaction included as fixed effects, while individual animals were considered random effects nested within treatment groups. When the treatment × time interaction was significant, simple effect tests were performed; when the interaction was not significant, only the main effects were evaluated. Post hoc multiple comparisons were conducted using the Sidak adjustment method. Data are presented as mean ± standard error of the mean (SEM). Differences were considered statistically significant at *p* < 0.05, while 0.05 ≤ *p* < 0.10 was interpreted as a numerical trend.

## 3. Results

### 3.1. Production Performance

The growth performance data of beef cattle are presented in Table 2. Dietary RP-GABA supplementation had no significant effects on initial body weight, final body weight, or ADG of beef cattle. A numerical trend was observed for ADG among dietary treatments (*p* = 0.099).

### 3.2. Breathing Rate and Temperature

The physiological parameters of beef cattle in each group are presented in Table 3. Time had significant effects on body temperature and breathing rate (all *p* < 0.001). Compared with Day 21, breathing rate was significantly increased on Days 23 (*p* = 0.002) and 54 (*p* < 0.001); however, no significant difference was observed between Day 23 and Day 54. Compared with Day 23, body temperature was significantly decreased on Day 54 (*p* < 0.001). Dietary RP-GABA supplementation had no significant effect on body temperature or breathing rate.

The interaction between dietary RP-GABA supplementation and time significantly affected breathing rate (*p* = 0.005). Specifically, dietary RP-GABA supplementation attenuated the transport-induced increase in breathing rate on Day 23.

### 3.3. Serum Biochemical Parameters

The serum biochemical parameters of beef cattle in each group are presented in Table 4. Time had a highly significant effect on all serum biochemical parameters (all *p* < 0.001). Compared with Day 21, the concentrations of ALT, AST, LDH, CK, ALB, TC, UREA, CREA, TP, LDL-C, HDL-C, and LPS were significantly increased on Day 23 (all *p* < 0.001), whereas TG (*p* = 0.005) and GLU (*p* < 0.001) concentrations were significantly decreased. Compared with Day 23, the concentrations of ALT (*p* < 0.001), AST (*p* < 0.001), ALP (*p* < 0.001), LDH (*p* < 0.001), CK (*p* < 0.001), ALB (*p* < 0.001), TC (*p* < 0.001), TG (*p* = 0.011), UREA (*p* < 0.001), LDL-C (*p* < 0.001), HDL-C (*p* < 0.001), and HIS (*p* < 0.001) were significantly decreased on Day 54, whereas GLU (*p* = 0.026) concentration was significantly increased. Dietary RP-GABA supplementation significantly affected ALP (*p* = 0.042), TP (*p* = 0.035), TC (*p* = 0.034), LDL-C (*p* = 0.023), GLU (*p* = 0.025), and HIS (*p* = 0.033) concentrations. ALP concentration in the G-M group was significantly lower than that in the CON group (*p* = 0.005). Only the G-H group exhibited a significantly higher TP concentration than the CON group (*p* = 0.006). Compared with the CON group, the G-L group showed significantly lower TC and LDL-C concentrations (*p* = 0.006; *p* = 0.006). GLU concentration in the G-M group was significantly lower than those in the CON and G-H groups (*p* = 0.004; *p* = 0.019), but showed no significant difference compared with the G-L group. HIS concentration in the G-L group was significantly higher than that in the G-M and G-H groups (*p* = 0.033; *p* = 0.006), while no significant difference was observed compared with the CON group.

The interaction between dietary RP-GABA supplementation and time significantly affected ALB and GLU concentrations (*p* = 0.032; *p* = 0.002). Compared with the CON group, the G-L (*p* = 0.002), G-M (*p* = 0.018) and G-H (*p* < 0.001) groups attenuated the transport-induced increase in ALB concentration on Day 54. In contrast, the G-M group showed a lower GLU concentration than the CON group only on Day 21 (*p* < 0.001).

In summary, long-distance transport significantly altered serum biochemical parameters in beef cattle, followed by a gradual recovery over time, except for ALP, TG, and CREA. Different doses of RP-GABA produced different effects: the G-L group primarily reduced serum TC and LDL-C concentrations; the G-M group reduced serum ALP levels; and the G-H group increased serum TP levels.

### 3.4. Serum Hormone Levels

The hormone levels in each group of beef cattle are presented in Table 5. Time had a significant effect on all hormone concentrations (all *p* < 0.001). Compared with Day 21, serum COR concentration was significantly increased (*p* = 0.027), whereas T3 concentration was significantly decreased on Day 23 (*p* < 0.001); no significant changes were observed in the concentrations of the other hormones. On Day 54, the concentrations of COR, ACTH, T3, T4, and EPI were significantly lower than those on Day 23 (all *p* < 0.001). Dietary RP-GABA supplementation significantly affected serum T3 and T4 concentrations (*p* < 0.001; *p* = 0.003). The T3 and T4 concentrations in the G-M group were significantly higher than those in the CON (*p* < 0.001; *p* =0.003) and G-H (*p* < 0.001; *p* =0.001) groups, and T3 concentration in the G-L group was significantly higher than that in the G-M group (*p* = 0.030).

The interaction between dietary RP-GABA supplementation and time had significant effects on COR, T3, T4, and EPI concentrations (all *p* < 0.001). Compared with the CON group, the G-H group showed lower COR and EPI concentrations after long-distance transport (Day 23) (all *p* < 0.001). The G-L group attenuated the transport-induced declines in T3 and T4 concentrations on Day 23.

In summary, long-distance transport significantly increased serum COR concentration and decreased T3 concentration in beef cattle. On Day 54, serum concentrations of COR, ACTH, T3, T4, and EPI were decreased compared with Day 23. Regarding the effects of RP-GABA supplementation, the G-L group alleviated the transport-induced decreases in T3 and T4 concentrations, the G-M group generally increased IL-4 concentrations, and the G-H group reduced COR and EPI concentrations on Day 23.

### 3.5. Serum Immune Parameters

The serum immune parameters in each group of beef cattle are presented in Table 6. Time had a significant effect on IL-1β (*p* = 0.004), IL-4 (*p* < 0.001), IL-6 (*p* < 0.001), IL-10 (*p* = 0.037) and TNF-α concentrations (*p* < 0.001). Compared with Day 21, the concentrations of IL-4, IL-6, and TNF-α were significantly increased on Day 23 (all *p* < 0.001). On Day 54, IL-4 and TNF-α concentrations showed no significant differences compared with Day 23 but remained significantly higher than those on Day 21 (all *p* < 0.001). IL-6 concentration on Day 23 was significantly higher than those on Days 21 and 54 (all *p* < 0.001), whereas no significant difference was observed between Days 21 and 54. IL-10 concentration on Day 21 was significantly higher than that on Day 54 (*p* = 0.011), with no significant difference compared with Day 23. Compared with Day 21, IL-1β concentration did not change significantly on Day 23 but was significantly higher than that on Day 54 (*p* = 0.001). Dietary RP-GABA supplementation significantly affected serum IL-4 and TNF-α concentrations (*p* = 0.012; *p* = 0.037). IL-4 concentrations in the G-M and G-H groups were significantly higher than those in the CON group (*p* = 0.006; *p* = 0.007), whereas no significant differences were observed between these groups and the G-L group. TNF-α concentration in the G-L group was significantly lower than those in the CON and G-H groups (*p* = 0.008; *p* = 0.018), but showed no significant difference compared with the G-M group.

The interaction between dietary RP-GABA supplementation and time significantly affected serum IL-4 (*p* = 0.007), IL-6 (*p* = 0.008), and TNF-α (*p* < 0.001) concentrations. Compared with the CON group, the G-M and G-H groups maintained higher IL-4 concentrations after transport (Day 23), whereas the G-H group maintained higher IL-6 concentrations before and after long-distance transport (Days 21 and 54). In addition, RP-GABA supplementation attenuated the transport-induced increase in TNF-α concentration compared with the CON group on Day 23.

In summary, long-distance transport significantly increased serum IL-4, IL-6, and TNF-α concentrations, followed by a decrease in IL-1β and IL-6 concentrations during the recovery period. Dietary RP-GABA supplementation reduced TNF-α concentration, while the G-M and G-H groups showed higher IL-4 concentrations.

### 3.6. Serum Antioxidant Parameters

The serum antioxidant parameters in each group of beef cattle are presented in Table 7. Time had a significant effect on MDA (*p* < 0.001), T-AOC (*p* = 0.008), SOD (*p* < 0.001), CAT (*p* < 0.001) and GSH-Px (*p* < 0.001) concentrations. Compared with Day 21, serum MDA concentration was significantly increased on Day 23 (*p* < 0.001). On Day 23, the concentrations of T-AOC (*p* = 0.013; *p* = 0.004), CAT (all *p* < 0.001), SOD (*p* < 0.001; *p* = 0.026), and GSH-Px (all *p* < 0.001) were significantly higher than those on Days 21 and 54. Specifically, the concentrations of SOD and GSH-Px on Day 54 were significantly higher than those on Day 21 (*p* < 0.001; *p* = 0.016), whereas CAT concentration on Day 54 was significantly lower than that on Day 21 (*p* = 0.008). Dietary RP-GABA supplementation significantly affected serum T-AOC, SOD, and GSH-Px concentrations (all *p* < 0.001). The T-AOC concentration in the G-H group was significantly higher than those in the CON (*p* = 0.006), G-L (*p* < 0.001), and G-M (*p* < 0.001) groups, whereas no significant differences were observed among the CON, G-L, and G-M groups. SOD concentrations in the G-L (*p* = 0.036), G-M (*p* < 0.001), and G-H (*p* < 0.001) groups were significantly lower than those in the CON group, and the G-L group exhibited significantly higher SOD concentration than the G-M (*p* = 0.002) and G-H (*p* < 0.001) groups. The GSH-Px concentrations in the G-H and G-M groups were significantly higher than those in the CON (all *p* < 0.001) and G-L (*p* < 0.001; *p* = 0.004) groups.

The interaction between dietary RP-GABA supplementation and time had significant effects on serum T-AOC (*p* = 0.004), CAT (*p* = 0.045), and GSH-Px (*p* = 0.043) concentrations. Compared with the CON group, the G-H group showed higher T-AOC concentrations before and after transport (Days 21 and 23), whereas the G-L and G-H groups attenuated the transport-induced increase in CAT concentration on Day 23. Moreover, the G-M and G-H groups showed higher GSH-Px concentrations than the CON group after transport (Day 23) (*p* = 0.012; *p* < 0.001).

In summary, long-distance transport significantly increased the concentrations of MDA, T-AOC, CAT, SOD, and GSH-Px, followed by a subsequent decline. Different doses of RP-GABA produced different effects: the G-M and G-H groups increased GSH-Px concentrations, whereas only the G-H group increased T-AOC concentration.

## 4. Discussion

The results of this study indicate that RP-GABA supplementation did not significantly affect growth performance in beef cattle. Although ADG varied among dietary treatments, these differences were not statistically significant (*p* = 0.099). Similar findings have been reported in previous studies [20,21]. In conjunction with blood parameters, the higher T3 concentration in the G-L group may reflect a relatively active anabolic state, whereas the lower GLU and T3 concentrations in the G-M group may indicate alterations in energy metabolism and endocrine status following transport [22,23]. The higher EPI and COR concentrations, together with lower T3 and T4 concentrations, in the G-H group suggest greater alterations in stress-related endocrine responses [22,24]. These physiological changes may reflect differences in metabolic and endocrine responses among supplementation levels, which may be associated with variations in growth performance.

In the present study, transport significantly increased serum COR concentration in the CON group, indicating the activation of a physiological stress response. Elevated COR concentrations may reflect enhanced metabolic activity and physiological arousal, potentially contributing to increased oxygen demand and respiratory activity after transport [25,26]. Dietary RP-GABA supplementation alleviated the transport-induced increase in breathing rate. This effect may be associated with the inhibitory role of GABA in central nervous system excitability through GABA-A receptor activation, thereby contributing to the regulation of respiratory responses [27,28,29].

On the one hand, GABA can interact with GABA receptors and suppress the TLR4/NF-κB signaling pathway in the liver, thereby reducing the expression of pro-inflammatory cytokines. On the other hand, GABA can increase the mRNA expression of glutathione peroxidase 1, heme oxygenase-1, and SOD in the liver, thereby enhancing hepatic antioxidant capacity and exerting hepatoprotective effects [30,31]. In the present study, ALP concentration was significantly decreased in the G-M group in beef cattle, suggesting an improvement in liver function, which is consistent with the findings reported by Su et al. [32]. Meanwhile, TC and LDL-C concentrations in the G-L group were significantly lower than those in the CON group. It is speculated that circulating lipids may be redistributed to meet energy requirements and tissue repair processes during stress recovery. The increased utilization of circulating lipids may have subsequently triggered a compensatory increase in serum lipid concentrations [33]. In this study, TP concentration in the G-H group was significantly higher than that in the CON group, whereas ALB concentration did not differ significantly among groups. This suggests that the increase in TP may have been attributed to changes in other protein fractions, such as globulins (e.g., IgM and IgA), which is consistent with previous studies involving GABA supplementation [12,34,35]. Furthermore, dietary RP-GABA supplementation promoted the recovery of ALB concentration on Day 54, which may contribute to the recovery of fluid balance after transport. However, dietary RP-GABA supplementation further reduced serum GLU concentration in beef cattle, which differs from previous findings [24]. This may be related to the higher T3 concentrations observed after RP-GABA supplementation, as thyroid hormones regulate glucose metabolism and tissue glucose utilization [24,36,37]. In addition, GABA can enhance the expression of tight junction proteins, including Claudin-1, Claudin-2, and ZO-1, thereby maintaining intestinal barrier integrity [38,39]. This mechanism may explain the lower HIS concentrations observed in the G-M and G-H groups compared with the CON group.

GABA may exert regulatory effects through GABA-A receptor signaling, which induces neuronal hyperpolarization and may reduce the activity of stress-related neurons, thereby attenuating HPA-axis activation and glucocorticoid secretion [40,41]. This mechanism may partly explain the reduced COR concentrations observed in the G-H group in the present study, which is consistent with previous findings by Li et al. [41]. As an inhibitory neurotransmitter, GABA may also regulate sympathetic nervous system activity and influence EPI secretion [42]. In the present study, the G-H group showed elevated EPI and COR concentrations on Day 21 but lower concentrations on Day 23 compared with the CON group. This response pattern may indicate a stress preconditioning effect induced by high-dose RP-GABA supplementation. The elevated pre-transport EPI and COR concentrations may reflect transient activation of the HPA and sympathetic–adrenal–medullary axis, representing a pre-activated state induced by high-dose RP-GABA supplementation, which is similar to the findings reported by Zhang et al. [14]. This pre-activated state may enhance the feedback regulation of stress-responsive systems, contributing to the rapid attenuation of stress hormone responses after transport [43,44]. However, this hypothesis requires further validation. In addition to regulating stress responses, GABA may influence thyroid hormone metabolism and contribute to the maintenance of endocrine homeostasis under stressful conditions. In the present study, RP-GABA supplementation increased T3 and T4 concentrations, indicating a potential dose-dependent response with an initial increase followed by a decline. The elevated thyroid hormone concentrations in the G-L group may be associated with improved endocrine adaptation and enhanced peripheral conversion of T4 to T3, whereas the reduced response at higher supplementation levels may reflect feedback regulation of thyroid hormone metabolism [45,46]. These findings suggest that RP-GABA may contribute to transport stress adaptation by modulating endocrine homeostasis, although the underlying mechanisms require further investigation.

Previous studies have demonstrated that GABA can suppress the production of pro-inflammatory cytokines through GABA receptors and transporters (such as GAT2) and maintain immune homeostasis by inhibiting NF-κB signaling [47,48]. In the present study, IL-4 concentrations in the G-M and G-H groups were higher than those in the CON group both before and after transport, which differs from the findings of previous studies [49]. Based on previous studies and the observed changes in inflammatory cytokines, the higher IL-4 concentrations in the G-M and G-H groups may reflect adaptive immune regulation during transport stress [49,50]. Previous studies have shown that GABA may exert anti-inflammatory effects by regulating the HPA axis, glucocorticoid release, and NF-κB signaling, thereby modulating the production of pro-inflammatory cytokines such as IL-6 [51,52,53]. In the present study, the reduced COR concentration and the absence of further elevation in IL-6 after transport in the G-H group suggest that high-dose RP-GABA may contribute to the regulation of neuroendocrine–immune interactions during transport stress. However, the specific relationship between glucocorticoid regulation and IL-6 responses requires further investigation. Cui et al. [54] reported that GABA can inhibit TLR4/NF-κB pathway activation in hepatic Kupffer cells, thereby reducing TNF-α release. In the present study, RP-GABA supplementation prevented the transport-induced increase in TNF-α concentration, consistent with previous reports that GABA may modulate inflammatory responses through TLR4/NF-κB-related pathways. Overall, RP-GABA supplementation may contribute to the regulation of inflammatory responses during transport stress; however, its effects on individual cytokines and the underlying mechanisms require further investigation.

Reactive oxygen species (ROS) generated under stress conditions can attack polyunsaturated fatty acids in cell membranes, resulting in the formation of MDA, which is widely used as an indicator of oxidative damage in beef cattle [55]. In the present study, serum MDA concentration significantly increased after long-distance transport, consistent with the findings of Mao et al. [55], indicating that cattle experienced transport-induced oxidative stress. Under oxidative stress conditions, SOD serves as the first line of antioxidant defense by catalyzing the dismutation of O_2_^−^ into H_2_O_2_ and O_2_, while CAT and GSH-Px contribute to the removal of H_2_O_2_ and maintenance of redox homeostasis [56]. Although antioxidant enzyme activities may decrease due to excessive consumption under severe oxidative stress, the activities of SOD, CAT, GSH-Px, and T-AOC significantly increased after transport in the present study. Combined with the elevated MDA concentration, these results suggest that transport activated the antioxidant defense system of cattle, leading to a compensatory increase in antioxidant enzyme activities and total antioxidant capacity rather than an improvement in oxidative status. Previous studies have shown that GABA may enhance antioxidant defense by regulating Nrf2-related signaling pathways and promoting the expression of downstream antioxidant enzymes, including HO-1, NQO1, SOD, CAT, and GSH-Px [57]. This antioxidant effect has been observed in various animal models; for example, Fathi et al. [56] reported that GABA supplementation increased SOD, CAT, and GSH-Px activities in stressed broiler chickens. In the present study, GSH-Px activity in the G-M and G-H groups on Day 23 was significantly higher than that in the CON and G-L groups, and T-AOC levels in the G-H group were higher than those in the other groups on Days 21 and 23. These findings suggest that, although transport induced a compensatory antioxidant response, RP-GABA supplementation may further modulate antioxidant defense capacity during transport stress. However, dietary RP-GABA supplementation decreased SOD activity in the present study, which may be associated with changes in antioxidant defense regulation and reduced oxidative pressure following RP-GABA supplementation [58].

Previous anti-stress additives, such as vitamin E, selenium, and yeast culture, have primarily exerted their beneficial effects through modulation of rumen fermentation, immune function, and antioxidant capacity [59,60]. In contrast, our findings suggest that dietary RP-GABA supplementation may alleviate transport stress by regulating stress-related hormone secretion, enhancing antioxidant capacity, and modulating immune function. In addition, different supplementation levels exerted distinct effects on these physiological responses. However, this study has some limitations. The absence of a positive control group receiving conventional anti-stress additives prevented direct comparisons between RP-GABA and other anti-stress strategies. In addition, the lack of baseline physiological measurements before the experiment limited the comprehensive evaluation of the dynamic regulatory effects of RP-GABA during the feeding and transport stress periods. Although strict feeding management was implemented to ensure RP-GABA supplementation, RP-GABA was incorporated into the concentrate, and individual differences in concentrate intake may have resulted in slight variations in actual RP-GABA intake among cattle. Future studies incorporating long-term monitoring and comparisons with other anti-stress interventions are warranted to further elucidate the potential mechanisms underlying the role of RP-GABA in alleviating transport stress in beef cattle.

## 5. Conclusions

Long-distance transport stress impaired antioxidant status, immune function, and stress-related hormone secretion in beef cattle. Dietary RP-GABA supplementation alleviated transport-induced stress by regulating endocrine responses, enhancing antioxidant defense, and modulating inflammatory responses. Different doses resulted in distinct physiological responses. The G-L group showed relatively limited effects on stress-related hormones, immune function, and antioxidant capacity, whereas the G-H group exhibited greater alterations in stress-related hormones, antioxidant status, and immune parameters, but had higher pre-transport stress hormone concentrations. The G-M group exhibited a relatively balanced regulatory effect across multiple physiological parameters, characterized by higher T4, GSH-Px, and IL-4 concentrations and lower ALP and TNF-α concentrations. Therefore, under the conditions of this study, dietary supplementation with 500 mg/kg RP-GABA in concentrate may represent a potential level for maintaining balanced physiological responses in beef cattle.

## Figures and Tables

**Table 1:** Diet composition and nutrient levels (%, dry matter basis) ^1^.

Ingredients	Basal Ration	Nutrient Level	Basal Ration
Corn	17.8	DM, %	90.58
Wheat Bran	4.4	CP, %	11.65
Dried Sugar Beet Pulp	11.2	EE, %	4.25
Dried Brewer’s Grains	18.0	CF, %	16.92
NaCl	0.3	NDF, %	41.58
CaHPO_4_	0.1	ADF, %	25.39
CaCO_3_	0.6	ASH, %	6.23
Premix ^2^	2.4	Gross Heat, MJ/kg	15.55
Hay	45.20		
Total	100		

^1^ Dietary ingredients are calculated values; the nutritional levels of the diet are measured values. ^2^ The premix provides per kilogram of feed: Vitamin A 4400 IU, Vitamin D 600 IU, Vitamin E 120 IU, Iron 110 mg, Copper 10 mg, Manganese 40 mg, Zinc 60 mg, Selenium 0.3 mg, Iodine 0.5 mg, and Cobalt 0.1 mg.

**Table 2:** Effects of dietary supplementation with rumen-protected γ-aminobutyric acid (RP-GABA) on growth performance of beef cattle.

Item	Group ^1^				*p*-Value
	CON	G-L	G-M	G-H	
Initial body weight (kg)	183.00 ± 5.1	181.30 ± 5.0	181.33 ± 6.3	182.18 ± 7.1	0.997
Final body weight (kg)	220.42 ± 6.3	232.18 ± 9.7	216.30 ± 9.2	215.11 ± 10.2	0.550
Average daily gain (kg)	0.71 ± 0.08	0.96 ± 0.11	0.66 ± 0.08	0.62 ± 0.12	0.099

^1^ CON = control group without RP-GABA supplementation; G-L, G-M, and G-H = groups supplemented with 300, 500, and 700 mg/kg of RP-GABA based on concentrate dry matter, respectively.

**Table 3:** Effects of dietary rumen-protected γ-aminobutyric acid (RP-GABA) supplementation on breathing rate and temperature in beef cattle.

Diet ^1^	Time ^2^	Temperature (°C)	Breathing Rate (Times/Min)
	21 d	39.51 ± 0.14	42.60 ± 2.26 ^y^
CON	23 d	39.41 ± 0.06	53.17 ± 2.27 ^x^
	54 d	38.92 ± 0.04	52.33 ± 2.62 ^xab^
	21 d	39.36 ± 0.06	43.80 ± 2.65
G-L	23 d	39.37 ± 0.06	49.08 ± 3.23
	54 d	38.98 ± 0.05	42.91 ± 2.68 ^b^
	21 d	39.33 ± 0.05	41.18 ± 3.30 ^y^
G-M	23 d	39.44 ± 0.08	49.00 ± 1.93 ^xy^
	54 d	39.04 ± 0.08	54.42 ± 4.78 ^xab^
	21 d	39.46 ± 0.11	45.33 ± 2.89 ^y^
G-H	23 d	39.31 ± 0.13	44.83 ± 3.79 ^y^
	54 d	39.30 ± 0.20	59.45 ± 2.26 ^xa^
Main effect			
	CON	39.28 ± 0.12	49.76 ± 2.69
Diet	G-L	39.24 ± 0.08	45.42 ± 2.91
	G-M	39.27 ± 0.09	48.40 ± 3.78
	G-H	39.36 ± 0.15	49.60 ± 3.56
	21 d	39.41 ± 0.10 ^x^	43.28 ± 2.75 ^y^
Time	23 d	39.38 ± 0.09 ^x^	49.02 ± 2.94 ^x^
	54 d	39.05 ± 0.12 ^y^	52.33 ± 3.59 ^x^
*p*-value ^3^			
Diet		0.657	0.384
Time		<0.001	<0.001
Diet × Time		0.116	0.005

^1^ CON = control group without RP-GABA supplementation; G-L, G-M, and G-H = groups supplemented with 300, 500, and 700 mg/kg of RP-GABA based on concentrate dry matter, respectively. ^2^ “21 d” refers to the day before the beef cattle were transported; “23 d” refers to the day the beef cattle arrived; “54 d” refers to the 31st day after the beef cattle were transported. ^3^ “xy” indicates differences in data across time points, and “ab” indicates differences in data across treatment groups; if the letters differ, the difference is statistically significant (*p* < 0.05).

**Table 4:** Effects of dietary rumen-protected γ-aminobutyric acid (RP-GABA) supplementation on serum biochemical parameters in beef cattle.

**Diet ^1^**	**Time ^2^**	**ALT (U/L)**	**AST (U/L)**	**ALP (U/L)**	**LDH (U/L)**	**CK (U/L)**	**ALB (g/L)**	**TC (mmol/L)**	**TG (mmol/L)**
	21 d	101.17 ± 6.09	78.36 ± 7.78	116.48 ± 9.63	2725.51 ± 82.77	178.59 ± 12.92	27.10 ± 0.41 ^y^	1.91 ± 0.10	0.27 ± 0.03
CON	23 d	121.45 ± 7.78	113.73 ± 7.97	123.63 ± 12.98	3258.52 ± 123.26	700.60 ± 50.51	28.82 ± 0.58 ^x^	2.09 ± 0.12	0.23 ± 0.03
	54 d	78.32 ± 3.91	65.38 ± 2.21	74.73 ± 6.21	2624.76 ± 76.20	150.20 ± 10.97	28.21 ± 0.41 ^x^	1.60 ± 0.09	0.23 ± 0.01
	21 d	120.59 ± 12.64	117.05 ± 22.80	105.51 ± 10.60	2925.80 ± 123.45	223.99 ± 39.66	27.35 ± 0.53 ^y^	1.52 ± 0.09	0.24 ± 0.02
G-L	23 d	137.47 ± 14.54	135.14 ± 20.16	100.56 ± 9.59	3426.87 ± 98.29	539.70 ± 52.77	30.13 ± 0.40 ^x^	1.75 ± 0.10	0.22 ± 0.01
	54 d	81.89 ± 4.21	75.42 ± 5.01	73.57 ± 8.18	2730.41 ± 60.73	143.46 ± 10.44	28.46 ± 0.44 ^y^	1.30 ± 0.09	0.18 ± 0.01
	21 d	108.37 ± 9.12	99.78 ± 12.23	87.88 ± 9.32	2834.85 ± 66.48	215.31 ± 40.97	27.73 ± 0.59 ^y^	1.86 ± 0.14	0.27 ± 0.01
G-M	23 d	136.28 ± 8.41	129.69 ± 6.45	82.39 ± 6.79	3502.95 ± 80.56	735.64 ± 67.00	29.58 ± 0.51 ^x^	1.94 ± 0.13	0.23 ± 0.01
	54 d	82.11 ± 5.49	65.44 ± 2.21	60.35 ± 3.29	2808.98 ± 82.75	142.90 ± 10.67	28.25 ± 0.41 ^y^	1.61 ± 0.10	0.22 ± 0.02
	21 d	105.17 ± 7.25	75.55 ± 8.91	111.65 ± 9.82	2633.51 ± 72.55	162.53 ± 10.22	27.59 ± 0.37 ^y^	1.72 ± 0.08	0.25 ± 0.02
G-H	23 d	143.22 ± 10.72	136.59 ± 12.11	100.31 ± 8.42	3459.08 ± 107.90	592.24 ± 45.51	29.85 ± 0.44 ^x^	1.96 ± 0.09	0.25 ± 0.01
	54 d	77.56 ± 4.33	64.26 ± 3.29	67.04 ± 5.76	2627.94 ± 80.75	138.29 ± 17.63	27.38 ± 0.40 ^y^	1.41 ± 0.06	0.18 ± 0.02
Main effect									
Diet	CON	98.68 ± 7.88	84.51 ± 8.52	102.45 ± 11.54 ^a^	2855.20 ± 123.35	310.98 ± 75.94	28.08 ± 0.50	1.85 ± 0.12 ^a^	0.24 ± 0.02
	G-L	114.86 ± 13.15	110.86 ± 19.03	93.96 ± 10.10 ^ab^	3047.04 ± 129.10	304.83 ± 62.98	28.69 ± 0.56	1.53 ± 0.10 ^b^	0.21 ± 0.01
	G-M	109.69 ± 10.00	99.24 ± 11.02	77.35 ± 7.62 ^b^	3062.28 ± 121.17	380.38 ± 91.22	28.53 ± 0.55	1.80 ± 0.13 ^a^	0.24 ± 0.02
	G-H	109.54 ± 11.01	92.93 ± 12.78	93.74 ± 9.69 ^ab^	2914.81 ± 143.69	292.55 ± 66.69	28.30 ± 0.51	1.70 ± 0.10 ^ab^	0.23 ± 0.02
	21 d	108.83 ± 9.07 ^y^	92.69 ± 14.58 ^y^	105.38 ± 10.04 ^x^	2778.75 ± 92.49 ^y^	195.11 ± 29.46 ^y^	27.44 ± 0.47 ^z^	1.75 ± 0.11 ^y^	0.26 ± 0.02 ^x^
Time	23 d	134.40 ± 10.67 ^x^	128.61 ± 12.83 ^x^	102.14 ± 10.41 ^x^	3409.09 ± 104.38 ^x^	644.02 ± 58.17 ^x^	29.59 ± 0.49 ^x^	1.93 ± 0.11 ^x^	0.23 ± 0.02 ^y^
	54 d	79.80 ± 4.36 ^z^	67.40 ± 3.41 ^z^	69.51 ± 6.16 ^y^	2690.55 ± 76.56 ^y^	144.38 ± 12.27 ^z^	28.09 ± 0.42 ^y^	1.49 ± 0.09 ^z^	0.21 ± 0.02 ^z^
*p*-value ^3^									
Diet		0.377	0.191	0.042	0.083	0.123	0.325	0.034	0.246
Time		<0.001	<0.001	<0.001	<0.001	<0.001	<0.001	<0.001	<0.001
Diet × Time		0.269	0.116	0.306	0.274	0.088	0.032	0.581	0.167
**Diet ^1^**	**Time ^2^**	**UREA (mmol/L)**	**CREA (umol/L)**	**TP (g/L)**	**LDL-C (mmol/L)**	**HDL-C (mmol/L)**	**GLU (mmol/L)**	**LPS (mg/L)**	**HIS (mg/L)**
	21 d	3.62 ± 0.23	101.52 ± 4.44	55.98 ± 0.70	0.52 ± 0.04	1.18 ± 0.04	3.87 ± 0.14 ^xa^	3.31 ± 0.11	123.33 ± 9.45
CON	23 d	6.03 ± 0.51	112.66 ± 5.07	60.00 ± 1.05	0.60 ± 0.04	1.28 ± 0.06	2.49 ± 0.30 ^y^	4.18 ± 0.44	156.11 ± 15.38
	54 d	4.58 ± 0.23	128.65 ± 4.33	59.01 ± 0.79	0.40 ± 0.02	1.09 ± 0.05	2.72 ± 0.18 ^y^	4.26 ± 0.24	82.85 ± 7.79
	21 d	3.67 ± 0.22	106.41 ± 4.27	55.07 ± 1.01	0.42 ± 0.03	0.99 ± 0.05	3.45 ± 0.10 ^xa^	3.70 ± 0.35	139.56 ± 10.58
G-L	23 d	7.02 ± 0.37	126.16 ± 5.50	60.81 ± 1.19	0.48 ± 0.03	1.15 ± 0.05	2.46 ± 0.18 ^y^	3.93 ± 0.30	173.88 ± 13.00
	54 d	5.02 ± 0.17	121.53 ± 4.57	59.88 ± 1.03	0.34 ± 0.02	0.92 ± 0.05	2.48 ± 0.10 ^y^	4.60 ± 0.47	90.04 ± 9.42
	21 d	4.09 ± 0.31	101.31 ± 4.26	55.65 ± 1.28	0.51 ± 0.03	1.16 ± 0.08	2.85 ± 0.14 ^xb^	3.17 ± 0.20	123.09 ± 13.24
G-M	23 d	6.59 ± 0.28	115.68 ± 5.26	61.18 ± 1.39	0.53 ± 0.03	1.25 ± 0.07	2.11 ± 0.19 ^y^	3.56 ± 0.22	137.98 ± 8.76
	54 d	4.30 ± 0.22	116.38 ± 3.70	61.13 ± 0.98	0.40 ± 0.02	1.09 ± 0.05	2.56 ± 0.09 ^xy^	3.94 ± 0.34	75.55 ± 5.88
	21 d	3.56 ± 0.19	106.75 ± 4.83	57.65 ± 0.87	0.54 ± 0.02	1.06 ± 0.04	3.50 ± 0.14 ^xa^	3.50 ± 0.16	129.54 ± 24.48
G-H	23 d	6.59 ± 0.25	117.99 ± 6.75	63.90 ± 0.90	0.59 ± 0.03	1.26 ± 0.04	2.48 ± 0.27 ^y^	4.44 ± 0.33	118.26 ± 15.71
	54 d	4.51 ± 0.28	125.64 ± 5.64	62.91 ± 1.33	0.38 ± 0.02	0.97 ± 0.05	2.84 ± 0.21 ^y^	4.47 ± 0.18	68.12 ± 7.20
Main effect									
Diet	CON	4.76 ± 0.44	115.64 ± 5.57	58.43 ± 0.96 ^b^	0.50 ± 0.04 ^a^	1.18 ± 0.05	2.98 ± 0.27 ^a^	3.97 ± 0.31	115.64 ± 14.31 ^ab^
	G-L	5.29 ± 0.49	118.16 ± 5.32	58.61 ± 1.29 ^b^	0.42 ± 0.03 ^b^	1.03 ± 0.06	2.80 ± 0.19 ^ab^	4.06 ± 0.38	136.82 ± 14.78 ^a^
	G-M	5.01 ± 0.43	110.97 ± 4.80	59.27 ± 1.42 ^ab^	0.48 ± 0.03 ^ab^	1.17 ± 0.07	2.50 ± 0.17 ^b^	3.54 ± 0.27	113.26 ± 12.28 ^b^
	G-H	4.90 ± 0.44	116.54 ± 6.06	61.45 ± 1.30 ^a^	0.51 ± 0.03 ^a^	1.10 ± 0.05	2.94 ± 0.24 ^a^	4.13 ± 0.27	106.37 ± 18.66 ^b^
	21 d	3.74 ± 0.24 ^z^	104.00 ± 4.38 ^y^	56.09 ± 1.00 ^y^	0.50 ± 0.03 ^y^	1.10 ± 0.06 ^y^	3.42 ± 0.16 ^x^	3.42 ± 0.23 ^y^	129.25 ± 15.55 ^x^
Time	23 d	6.55 ± 0.38 ^x^	118.17 ± 5.69 ^x^	61.43 ± 1.19 ^x^	0.55 ± 0.03 ^x^	1.24 ± 0.05 ^x^	2.39 ± 0.24 ^z^	4.02 ± 0.33 ^x^	147.81 ± 14.43 ^x^
	54 d	4.60 ± 0.23 ^y^	123.62 ± 4.64 ^x^	60.56 ± 1.09 ^x^	0.38 ± 0.02 ^z^	1.02 ± 0.05 ^z^	2.66 ± 0.16 ^y^	4.31 ± 0.32 ^x^	79.52 ± 7.79 ^y^
*p*-value ^3^									
Diet		0.346	0.628	0.035	0.023	0.061	0.025	0.127	0.033
Time		<0.001	<0.001	<0.001	<0.001	<0.001	<0.001	<0.001	<0.001
Diet × Time		0.073	0.104	0.569	0.406	0.535	0.002	0.616	0.546

^1^ CON = control group without RP-GABA supplementation; G-L, G-M, and G-H = groups supplemented with 300, 500, and 700 mg/kg of RP-GABA based on concentrate dry matter, respectively. ^2^ “21 d” refers to the day before the beef cattle were transported; “23 d” refers to the day the beef cattle arrived; “54 d” refers to the 31st day after the beef cattle were transported. ^3^ “xyz” indicates differences in data across time points, and “ab” indicates differences in data across treatment groups; if the letters differ, the difference is statistically significant (*p* < 0.05). Abbreviations: ALT, alanine transaminase; AST, aspartate transaminase; ALP, alkaline phosphatase; LDH, lactate dehydrogenase; CK, creatine kinase; TP, total protein; ALB, albumin; UREA, urea; CREA, creatinine; GLU, glucose; TC, total cholesterol; TG, triglycerides; HDL-C, high-density lipoprotein cholesterol; LDL-C, low-density lipoprotein cholesterol; LPS, lipopolysaccharide; HIS, histamine.

**Table 5:** Effects of dietary rumen-protected γ-aminobutyric acid (RP-GABA) supplementation on serum hormone levels in beef cattle.

Diet ^1^	Time ^2^	COR (ng/mL)	ACTH (pg/mL)	T3 (ng/mL)	T4 (ng/mL)	EPI (pg/mL)
	21 d	49.46 ± 2.41 ^yb^	37.55 ± 0.95	371.50 ± 15.55 ^xc^	153.28 ± 6.36 ^yb^	37.75 ± 2.83 ^xyb^
CON	23 d	67.55 ± 3.48 ^xa^	41.87 ± 1.60	435.47 ± 22.54 ^xb^	188.39 ± 10.65 ^xa^	47.69 ± 3.26 ^x^
	54 d	41.28 ± 2.83 ^z^	30.36 ± 1.82	250.97 ± 24.47 ^y^	126.76 ± 7.63 ^z^	31.06 ± 2.68 ^y^
	21 d	52.68 ± 2.25 ^xab^	37.74 ± 1.62	632.61 ± 26.93 ^xa^	162.52 ± 10.23 ^yb^	42.56 ± 2.70 ^xyab^
G-L	23 d	63.11 ± 4.34 ^xa^	40.40 ± 2.30	680.74 ± 41.50 ^xa^	222.98 ± 9.04 ^xa^	44.59 ± 2.91 ^x^
	54 d	38.69 ± 2.36 ^y^	30.93 ± 1.44	245.46 ± 8.23 ^y^	124.90 ± 5.35 ^z^	32.98 ± 2.72 ^y^
	21 d	49.47 ± 2.87 ^yb^	35.11 ± 1.76	698.64 ± 25.46 ^xa^	218.45 ± 11.53 ^xa^	40.17 ± 3.45 ^xyb^
G-M	23 d	60.90 ± 1.91 ^xa^	41.39 ± 2.57	435.93 ± 23.65 ^yb^	189.97 ± 12.62 ^xa^	41.07 ± 3.41 ^x^
	54 d	43.62 ± 2.16 ^y^	31.05 ± 1.45	269.06 ± 12.27 ^z^	129.49 ± 4.23 ^y^	31.63 ± 2.64 ^y^
	21 d	61.49 ± 3.55 ^xa^	39.59 ± 2.73	514.81 ± 19.67 ^xb^	188.06 ± 10.96 ^xab^	54.64 ± 4.62 ^xa^
G-H	23 d	41.58 ± 4.10 ^yb^	34.12 ± 1.88	235.22 ± 26.25 ^yc^	125.14 ± 6.78 ^yb^	38.12 ± 4.43 ^y^
	54 d	40.58 ± 1.61 ^y^	28.16 ± 2.09	307.11 ± 18.55 ^y^	146.83 ± 6.53 ^y^	26.09 ± 2.48 ^z^
Main effect						
Diet	CON	52.00 ± 2.41	36.11 ± 2.08	344.75 ± 31.26 ^c^	154.06 ± 11.13 ^bc^	38.29 ± 3.50
	G-L	52.17 ± 2.25	36.62 ± 2.14	531.69 ± 62.95 ^a^	172.86 ± 14.49 ^ab^	40.37 ± 3.07
	G-M	49.47 ± 2.87	35.98 ± 2.30	473.56 ± 55.62 ^b^	180.73 ± 14.66 ^a^	37.79 ± 3.34
	G-H	61.49 ± 3.55	34.12 ± 2.58	353.67 ± 41.04 ^c^	153.53 ± 11.24 ^c^	40.00 ± 5.16
	21 d	53.28 ± 3.08 ^y^	37.50 ± 1.87 ^x^	554.39 ± 42.27 ^x^	180.58 ± 12.15 ^x^	43.78 ± 3.87 ^x^
Time	23 d	58.57 ± 4.52 ^x^	39.51 ± 2.24 ^x^	451.29 ± 54.35 ^y^	182.58 ± 14.11 ^x^	43.00 ± 3.58 ^x^
	54 d	41.07 ± 2.32 ^z^	30.15 ± 1.71 ^y^	266.40 ± 18.74 ^z^	131.46 ± 6.54 ^y^	30.50 ± 2.66 ^y^
*p*-value ^3^						
Diet		0.217	0.297	<0.001	0.003	0.806
Time		<0.001	<0.001	<0.001	<0.001	<0.001
Diet × Time		<0.001	0.104	<0.001	<0.001	<0.001

^1^ CON = control group without RP-GABA supplementation; G-L, G-M, and G-H = groups supplemented with 300, 500, and 700 mg/kg of RP-GABA based on concentrate dry matter, respectively. ^2^ “21 d” refers to the day before the beef cattle were transported; “23 d” refers to the day the beef cattle arrived; “54 d” refers to the 31st day after the beef cattle were transported. ^3^ “xyz” indicates differences in data across time points, and “abc” indicates differences in data across treatment groups; if the letters differ, the difference is statistically significant (*p* < 0.05). Abbreviations: COR, cortisol; ACTH, adrenocorticotropic hormone; T3, triiodothyronine; T4, thyroxine; EPI, epinephrine.

**Table 6:** Effects of dietary rumen-protected γ-aminobutyric acid (RP-GABA) supplementation on serum immune parameters in beef cattle.

Diet ^1^	Time ^2^	IL-1β (pg/mL)	IL-4 (pg/mL)	IL-6 (pg/mL)	IL-10 (ng/mL)	TNF-α (pg/mL)
	21 d	402.08 ± 65.15	482.39 ± 41.58 ^yb^	132.32 ± 34.21 ^yab^	5.19 ± 0.72	68.81 ± 10.25 ^y^
CON	23 d	488.64 ± 52.15	682.75 ± 127.30 ^yb^	190.53 ± 20.96 ^x^	4.59 ± 0.55	138.07 ± 15.77 ^xa^
	54 d	237.71 ± 51.29	1785.55 ± 245.13 ^x^	135.82 ± 27.22 ^yab^	3.77 ± 0.48	86.97 ± 6.36 ^y^
	21 d	338.34 ± 59.35	872.04 ± 140.66 ^yab^	102.09 ± 24.53 ^yb^	3.38 ± 0.76	47.11 ± 3.51
G-L	23 d	417.75 ± 76.79	1088.32 ± 156.72 ^xyb^	245.04 ± 32.91 ^x^	2.41 ± 0.41	59.74 ± 5.84 ^b^
	54 d	255.24 ± 47.28	2103.50 ± 325.89 ^x^	119.48 ± 27.13 ^yb^	3.25 ± 0.75	65.03 ± 4.42
	21 d	434.97 ± 58.51	1183.58 ± 168.00 ^yab^	100.42 ± 15.90 ^yb^	5.24 ± 0.88	47.74 ± 6.76 ^y^
G-M	23 d	480.91 ± 80.66	2493.12 ± 200.44 ^xa^	300.68 ± 20.22 ^x^	3.29 ± 0.35	48.44 ± 4.31 ^yb^
	54 d	275.17 ± 77.73	2033.84 ± 209.81 ^xy^	86.44 ± 12.51 ^yb^	4.14 ± 0.66	127.65 ± 23.37 ^x^
	21 d	399.48 ± 79.11	1569.34 ± 183.89 ^ya^	202.22 ± 28.70 ^a^	6.12 ± 1.70	75.87 ± 10.76
G-H	23 d	415.97 ± 109.74	2235.18 ± 213.58 ^xa^	239.95 ± 59.24	6.58 ± 1.34	91.06 ± 14.70 ^ab^
	54 d	351.68 ± 82.52	1698.00 ± 456.97 ^xy^	228.79 ± 37.29 ^a^	3.93 ± 0.83	107.72 ± 17.77
Main effect						
Diet	CON	348.49 ± 61.91	1008.62 ± 231.58 ^b^	151.47 ± 28.16	4.47 ± 0.59	98.50 ± 13.90 ^a^
	G-L	341.72 ± 64.51	1213.50 ± 229.18 ^ab^	160.15 ± 33.78	3.02 ± 0.65	57.07 ± 5.04 ^b^
	G-M	401.86 ± 74.43	1793.49 ± 247.92 ^a^	162.94 ± 32.81	4.34 ± 0.72	74.61 ± 17.59 ^ab^
	G-H	387.87 ± 86.64	1827.03 ± 286.48 ^a^	223.51 ± 42.81	5.72 ± 1.36	91.53 ± 14.56 ^a^
	21 d	393.23 ± 62.91 ^x^	1048.87 ± 177.88 ^y^	134.31 ± 28.35 ^y^	4.99 ± 1.08 ^x^	60.04 ± 8.85 ^y^
Time	23 d	449.79 ± 77.93 ^x^	1535.93 ± 270.75 ^x^	244.00 ± 37.78 ^x^	4.43 ± 0.94 ^xy^	87.15 ± 15.35 ^x^
	54 d	272.93 ± 62.81 ^y^	1894.34 ± 296.50 ^x^	143.94 ± 30.71 ^y^	3.80 ± 0.65 ^y^	97.49 ± 15.69 ^x^
*p*-value ^3^						
Diet		0.825	0.012	0.155	0.099	0.037
Time		0.004	<0.001	<0.001	0.037	<0.001
Diet × Time		0.954	0.007	0.008	0.561	<0.001

^1^ CON = control group without RP-GABA supplementation; G-L, G-M, and G-H = groups supplemented with 300, 500, and 700 mg/kg of RP-GABA based on concentrate dry matter, respectively. ^2^ “21 d” refers to the day before the beef cattle were transported; “23 d” refers to the day the beef cattle arrived; “54 d” refers to the 31st day after the beef cattle were transported. ^3^ “xy” indicates differences in data across time points, and “ab” indicates differences in data across treatment groups; if the letters differ, the difference is statistically significant (*p* < 0.05). Abbreviations: IL-1β, interleukin-1β; IL-4, interleukin-4; IL-6, interleukin-6; IL-10, interleukin-10; TNF-α, tumor necrosis factor-alpha.

**Table 7:** Effects of dietary rumen-protected γ-aminobutyric acid (RP-GABA) supplementation on serum antioxidant parameters in beef cattle.

Diet ^1^	Time ^2^	MDA (nmol/mL)	T-AOC (mM)	SOD (U/mL)	CAT (U/mL)	GSH-Px (U/mL)
	21 d	2.00 ± 0.31	0.18 ± 0.02 ^yc^	15.42 ± 0.39	0.99 ± 0.14 ^y^	81.59 ± 12.28
CON	23 d	3.04 ± 0.21	0.30 ± 0.03 ^xab^	19.23 ± 0.53	1.78 ± 0.40 ^x^	141.16 ± 20.33 ^b^
	54 d	2.66 ± 0.23	0.25 ± 0.03 ^xy^	18.25 ± 0.81	0.94 ± 0.10 ^y^	110.79 ± 18.57
	21 d	2.14 ± 0.36	0.26 ± 0.02 ^ab^	14.83 ± 0.52	1.02 ± 0.14	111.25 ± 13.07
G-L	23 d	2.58 ± 0.45	0.22 ± 0.01 ^c^	18.83 ± 0.42	1.19 ± 0.17	147.23 ± 15.37 ^b^
	54 d	2.47 ± 0.26	0.20 ± 0.01	16.25 ± 0.44	0.97 ± 0.15	101.60 ± 11.99
	21 d	1.78 ± 0.30	0.22 ± 0.02 ^bc^	13.46 ± 0.44	0.96 ± 0.10 ^y^	105.17 ± 21.53 ^y^
G-M	23 d	3.53 ± 0.44	0.24 ± 0.01 ^bc^	16.79 ± 0.61	1.95 ± 0.27 ^x^	253.08 ± 25.80 ^xa^
	54 d	3.09 ± 0.45	0.22 ± 0.02	14.99 ± 0.38	0.61 ± 0.11 ^y^	174.41 ± 35.91 ^xy^
	21 d	2.76 ± 0.36	0.30 ± 0.02 ^xya^	12.36 ± 0.50	1.30 ± 0.15 ^x^	114.54 ± 16.17 ^y^
G-H	23 d	2.92 ± 0.32	0.34 ± 0.03 ^xa^	14.74 ± 0.97	1.37 ± 0.26 ^x^	269.94 ± 17.98 ^xa^
	54 d	2.86 ± 0.25	0.26 ± 0.01 ^y^	16.09 ± 0.82	0.63 ± 0.12 ^y^	178.95 ± 17.56 ^y^
Main effect						
Diet	CON	2.60 ± 0.26	0.25 ± 0.03 ^b^	17.73 ± 0.76 ^a^	1.17 ± 0.24	111.99 ± 18.41 ^b^
	G-L	2.40 ± 0.36	0.23 ± 0.01 ^b^	16.71 ± 0.67 ^b^	1.07 ± 0.15	122.03 ± 14.49 ^b^
	G-M	2.79 ± 0.45	0.23 ± 0.02 ^b^	15.08 ± 0.63 ^c^	1.17 ± 0.24	171.51 ± 32.83 ^a^
	G-H	2.84 ± 0.31	0.30 ± 0.02 ^a^	14.35 ± 0.88 ^c^	1.11 ± 0.21	200.32 ± 24.75 ^a^
	21 d	2.18 ± 0.34 ^y^	0.24 ± 0.02 ^y^	14.02 ± 0.57 ^z^	1.05 ± 0.13 ^y^	102.59 ± 16.22 ^z^
Time	23 d	3.00 ± 0.38 ^x^	0.27 ± 0.02 ^x^	17.46 ± 0.82 ^x^	1.54 ± 0.28 ^x^	199.59 ± 26.60 ^x^
	54 d	2.76 ± 0.30 ^x^	0.23 ± 0.02 ^y^	16.58 ± 0.74 ^y^	0.79 ± 0.12 ^z^	141.16 ± 24.39 ^y^
*p*-value ^3^						
Diet		0.204	<0.001	<0.001	0.713	<0.001
Time		<0.001	0.008	<0.001	<0.001	<0.001
Diet × Time		0.120	0.004	0.078	0.045	0.043

^1^ CON = control group without RP-GABA supplementation; G-L, G-M, and G-H = groups supplemented with 300, 500, and 700 mg/kg of RP-GABA based on concentrate dry matter, respectively. ^2^ “21 d” refers to the day before the beef cattle were transported; “23 d” refers to the day the beef cattle arrived; “54 d” refers to the 31st day after the beef cattle were transported. ^3^ “xyz” indicates differences in data across time points, and “abc” indicates differences in data across treatment groups; if the letters differ, the difference is statistically significant (*p* < 0.05). Abbreviations: MDA, malondialdehyde; T-AOC, total antioxidant capacity; GSH-Px, glutathione peroxidase; SOD, superoxide dismutase; CAT, catalase.

## Data Availability

Original data are available from the corresponding author upon reasonable request.

## References

[1] Padalino B. (2015). Effects of the Different Transport Phases on Equine Health Status, Behavior, and Welfare: A Review. J. Vet. Behav..

[2] Hong H., Lee E., Lee I.H., Lee S.R. (2018). Effects of Transport Stress on Physiological Responses and Milk Production in Lactating Dairy Cows. Asian-Australas. J. Anim. Sci..

[3] Li F., Shah A.M., Wang Z., Peng Q., Hu R., Zou H., Tan C., Zhang X., Liao Y., Wang Y. (2019). Effects of Land Transport Stress on Variations in Ruminal Microbe Diversity and Immune Functions in Different Breeds of Cattle. Animals.

[4] Nwe T.M., Hori E., Manda M., Watanabe S. (1996). Significance of Catecholamines and Cortisol Levels in Blood during Transportation Stress in Goats. Small Rumin. Res..

[5] Arthington J.D., Eicher S.D., Kunkle W.E., Martin F.G. (2003). Effect of Transportation and Commingling on the Acute-Phase Protein Response, Growth, and Feed Intake of Newly Weaned Beef Calves. J. Anim. Sci..

[6] van Dijk L.L., Siegmann S., Field N.L., Sugrue K., van Reenen C.G., Bokkers E.A.M., Sayers G., Conneely M. (2023). Effect of Source and Journey on Physiological Variables in Calves Transported by Road and Ferry between Ireland and the Netherlands. Front. Vet. Sci..

[7] Chirase N.K., Greene L.W., Purdy C.W., Loan R.W., Auvermann B.W., Parker D.B., Walborg E.F., Stevenson D.E., Xu Y., Klaunig J.E. (2004). Effect of Transport Stress on Respiratory Disease, Serum Antioxidant Status, and Serum Concentrations of Lipid Peroxidation Biomarkers in Beef Cattle. Am. J. Vet. Res..

[8] Earley B., Buckham Sporer K., Gupta S. (2017). Invited Review: Relationship between Cattle Transport, Immunity and Respiratory Disease. Animal.

[9] Uddin M.S., Schwartzkopf-Genswein K.S., Waldner M., Meléndez D.M., Niu Y.D., Alexander T.W. (2023). Auction Market Placement and a Rest Stop during Transportation Affect the Respiratory Bacterial Microbiota of Beef Cattle. Front. Microbiol..

[10] Xie S.W., Li Y.T., Zhou W.W., Tian L.X., Li Y.M., Zeng S.L., Liu Y.J. (2017). Effect of γ-Aminobutyric Acid Supplementation on Growth Performance, Endocrine Hormone and Stress Tolerance of Juvenile Pacific White Shrimp, Litopenaeus Vannamei, Fed Low Fishmeal Diet. Aquac. Nutr..

[11] Li M., Qiu L., Wang L., Wang W., Xin L., Li Y., Liu Z., Song L. (2016). The Inhibitory Role of γ-Aminobutyric Acid (GABA) on Immunomodulation of Pacific Oyster Crassostrea Gigas. Fish Shellfish Immunol..

[12] Chen J., Guo K., Song X., Lan L., Liu S., Hu R., Luo J. (2020). The Anti–Heat Stress Effects of Chinese Herbal Medicine Prescriptions and Rumen-Protected γ-Aminobutyric Acid on Growth Performance, Apparent Nutrient Digestibility, and Health Status in Beef Cattle. Anim. Sci. J..

[13] Cheng J.B., Bu D.P., Wang J.Q., Sun X.Z., Pan L., Zhou L.Y., Liu W. (2014). Effects of Rumen-Protected γ-Aminobutyric Acid on Performance and Nutrient Digestibility in Heat-Stressed Dairy Cows. J. Dairy Sci..

[14] Zhang Y.T., Yang Y., Bu D.P., Ma L. (2025). The Effects of Gamma-Aminobutyric Acid on Growth Performance, Diarrhoea, Ruminal Fermentation, and Antioxidant Capacity in Pre-Weaned Calves. Animal.

[15] Rackwitz R., Gäbel G. (2017). Gamma-Aminobutyric Acid (GABA) Permeates Ovine Ruminal and Jejunal Epithelia, Mainly by Passive Diffusion. J. Anim. Physiol. Anim. Nutr..

[16] Husnu G., Rochana A., Kamil K.A., Setyowati E. (2019). The Effect of Gamma Aminobutyric Acid and Ascorbic Acid on Bali Cattle Physiology During Transport. Pak. J. Nutr..

[17] Joo Y.H., Woo J.S., Lee H., Kim W.S., Park K.K., Choi Y. (2026). Effect of Dietary Supplementation with Rumen-Protected GABA (γ-Aminobutyric Acid) on Milk Productivity and Blood Profiles of Dairy Cattle Under Heat Stress Conditions. Animals.

[18] Dißmann L., Heinicke J., Jensen K.C., Amon T., Hoffmann G. (2022). How Should the Respiration Rate Be Counted in Cattle?. Vet. Res. Commun..

[19] Hine L., Laven R.A., Sahu S.K. (2015). An Analysis of the Effect of Thermometer Type and Make on Rectal Temperature Measurements of Cattle, Horses and Sheep. N. Z. Vet. J..

[20] Barido F.H., Lee C.W., Park Y.S., Kim D.Y., Lee S.K. (2021). The Effect of a Finishing Diet Supplemented with γ-Aminobutyric Acids on Carcass Characteristics and Meat Quality of Hanwoo Steers. Anim. Biosci..

[21] Bi C., Yin J., Yang W., Shi B., Shan A. (2020). Effects of Dietary γ-Aminobutyric Acid Supplementation on Antioxidant Status, Blood Hormones and Meat Quality in Growing-Finishing Pigs Undergoing Transport Stress. J. Anim. Physiol. Anim. Nutr..

[22] Kahl S., Bitman J. (1983). Relation of Plasma Thyroxine and Triiodothyronine to Body Weight in Growing Male and Female Holstein Cattle. J. Dairy Sci..

[23] Zeng H., Yin Y., Chen L., Xu Z., Luo Y., Wang Q., Yang B., Wang J. (2023). Alterations in Nutrient Digestion and Utilization Associated with Different Residual Feed Intake in Hu Sheep. Anim. Nutr..

[24] Guo K., Cao H., Zhu Y., Wang T., Hu G., Kornmatitsuk B., Luo J. (2018). Improving Effects of Dietary Rumen Protected γ-Aminobutyric Acid Additive on Apparent Nutrient Digestibility, Growth Performance and Health Status in Heat-Stressed Beef Cattle. Anim. Sci. J..

[25] Gupta S., Earley B., Crowe M.A. (2007). Effect of 12-Hour Road Transportation on Physiological, Immunological and Haematological Parameters in Bulls Housed at Different Space Allowances. Vet. J..

[26] Romero M.L., Butler L.K. (2007). Endocrinology of Stress. Int. J. Comp. Psychol..

[27] Eniolorunda O.O., Fashina O.E., Aro O.O. (2009). Respuesta Fisiológica de Adaptación de Bovinos Al Estrés Durante El Transporte de Larga Duración En Nigeria. Arch. Zootec..

[28] Schmid K., Bohmer G., Gebauer K. (1991). GABA(A) Receptor Mediated Fast Synaptic Inhibition in the Rabbit Brain-Stem Respiratory System. Acta Physiol. Scand..

[29] Jiang C., Chen Y., Sun T. (2025). From the Gut to the Brain, Mechanisms and Clinical Applications of γ-Aminobutyric Acid (GABA) on the Treatment of Anxiety and Insomnia. Front. Neurosci..

[30] Zhang S., Zhao J., Hu J., He H., Wei Y., Ji L., Ma X. (2022). Gama-Aminobutyric Acid (GABA) Alleviates Hepatic Inflammation via GABA Receptors/TLR4/NF-κB Pathways in Growing-Finishing Pigs Generated by Super-Multiparous Sows. Anim. Nutr..

[31] Chen Q., Hu D., Wu X., Feng Y., Ni Y. (2022). Dietary γ-Aminobutyric Acid Supplementation Inhibits High-Fat Diet-Induced Hepatic Steatosis via Modulating Gut Microbiota in Broilers. Microorganisms.

[32] Su Y., Cheng Z., Liu W., Wu T., Wang W., Lin M. (2023). Effects of Rumen-Protective γ-Aminobutyric Acid Additive on Lactation Performance and Serum Biochemistry in Heat-Stressed Cows. Front. Vet. Sci..

[33] Karlmar K.E. (1982). In Vitro Conversion of Cholesterol into Aldosterone and Cortisol in Different Zones of the Bovine Adrenal Cortex. Acta Endocrinol..

[34] Cheng J., Zheng N., Sun X., Li S., Wang J., Zhang Y. (2016). Feeding Rumen-Protected Gamma-Aminobutyric Acid Enhances the Immune Response and Antioxidant Status of Heat-Stressed Lactating Dairy Cows. J. Therm. Biol..

[35] Tang J., Chen Z. (2016). The Protective Effect of γ-Aminobutyric Acid on the Development of Immune Function in Chickens under Heat Stress. J. Anim. Physiol. Anim. Nutr..

[36] Zou Y., Yang Y., Chen W., Zhao X., Wan Z., Wang P., Chen J., Li Y., Song X. (2026). Creatine Pyruvate and Gamma-Aminobutyric Acid Mitigate Heat Stress in Goats: Mechanistic Insights from Antioxidant Function, Ruminal Epithelial Barrier Integrity, and Transcriptomics. J. Therm. Biol..

[37] Arneson A.G., Stewart J.W., Byrd M.K.H., Perry G.A., Rhoads M.L. (2024). Plasma γ-Aminobutyric Acid (GABA) Concentrations in Lactating Holstein Cows during Thermoneutral and Heat Stress Conditions and Their Relationships with Circulating Glucose, Insulin and Progesterone Levels. Vet. Sci..

[38] Wu X.Q., Li X.N., Cai F.K., Lin Y.J., Niu X.T., Chen X.M., Tong Y.N., Wang G.Q. (2025). Positive Effects of Gamma Aminobutyric Acid on Growth and Lipopolysaccharide-Induced Intestinal Mucosal Barrier Damage in Snakehead (*Channa argus*). Aquac. Int..

[39] Zeng Y., Hu H., He Y., Deng Z., Guo Y., Zhou X. (2024). Multi-Omics Reveal the Improvements of Nutrient Digestion, Absorption, and Metabolism and Intestinal Function via GABA Supplementation in Weanling Piglets. Animals.

[40] Mikkelsen J.D., Bundzikova J., Larsen M.H., Hansen H.H., Kiss A. (2008). GABA Regulates the Rat Hypothalamic-Pituitary-Adrenocortical Axis via Different GABA-A Receptor α-Subtypes. Ann. N. Y. Acad. Sci..

[41] Li Y.H., Li F., Liu M., Yin J.J., Cheng B.J., Shi B.M., Shan A.S. (2015). Effect of γ-Aminobutyric Acid on Growth Performance, Behavior and Plasma Hormones in Weaned Pigs. Can. J. Anim. Sci..

[42] Wible J.H., Luft F.C., DiMicco J.A. (1988). Hypothalamic GABA Suppresses Sympathetic Outflow to the Cardiovascular System. Am. J. Physiol. Regul. Integr. Comp. Physiol..

[43] Sabban E.L., Serova L.I. (2007). Influence of Prior Experience with Homotypic or Heterotypic Stressor on Stress Reactivity in Catecholaminergic Systems. Stress.

[44] Grissom N., Bhatnagar S. (2008). Habituation to Repeated Stress: Get Used to It. Neurobiol. Learn. Mem..

[45] Tapia-Arancibia L., Roussel J.P., Astier H. (1987). Evidence for a Dual Effect of γ-Aminobutyric Acid on Thyrotropin (TSH)-Induced TSH Release from Perifused Rat Pituitaries. Endocrinology.

[46] Jordan D., Poncet C., Veisseire M., Mornex R. (1983). Role of GABA in the Control of Thyrotropin Secretion in the Rat. Brain Res..

[47] Daskalova E., Tsibidakis H. (2026). Does GABA Supplementation Modulate Muscle Cytokine Expression and Inflammatory Status in Aged Rats?. Eur. J. Transl. Myol..

[48] Bhandage A.K., Jin Z., Korol S.V., Shen Q., Pei Y., Deng Q., Espes D., Carlsson P.O., Kamali-Moghaddam M., Birnir B. (2018). GABA Regulates Release of Inflammatory Cytokines From Peripheral Blood Mononuclear Cells and CD4+ T Cells and Is Immunosuppressive in Type 1 Diabetes. eBioMedicine.

[49] Chen S., Tan B., Xia Y., Liao S., Wang M., Yin J., Wang J., Xiao H., Qi M., Bin P. (2019). Effects of Dietary Gamma-Aminobutyric Acid Supplementation on the Intestinal Functions in Weaning Piglets. Food Funct..

[50] Li Y., Zhen S., Sun F., Cao L., Wang L. (2024). Effects of γ-Aminobutyric Acid on Growth Performance, Immunity, Antioxidant Capacity, and Intestinal Microbiota of Growing Minks. Vet. Sci..

[51] Cain D.W., Cidlowski J.A. (2017). Immune Regulation by Glucocorticoids. Nat. Rev. Immunol..

[52] Balcerowska M., Kwaśnik P. (2025). The Multifaceted Impact of Stress on Immune Function. Mol. Biol. Rep..

[53] Tong R.L., Kahn U.N., Grafe L.A., Hitti F.L., Fried N.T., Corbett B.F. (2023). Stress Circuitry: Mechanisms behind Nervous and Immune System Communication That Influence Behavior. Front. Psychiatry.

[54] Cui Q., Yang Q., Wang Z., Wan Y., Jiang D., Liao Y., Du W., Ding Y., Zheng J., Li X. (2025). GABA Ameliorates Diet-Induced Hepatic Steatosis and Insulin Resistance by Inhibiting Macrophage Activation. Diabetes Obes. Metab..

[55] Mao K., Lu G., Li Y., Zang Y., Zhao X., Qiu Q., Qu M., Ouyang K. (2022). Effects of Rumen-Protected Creatine Pyruvate on Blood Biochemical Parameters and Rumen Fluid Characteristics in Transported Beef Cattle. BMC Vet. Res..

[56] Fathi M., Saeedyan S., Kaoosi M. (2023). Gamma-Amino Butyric Acid (GABA) Supplementation Alleviates Dexamethasone Treatment-Induced Oxidative Stress and Inflammation Response in Broiler Chickens. Stress.

[57] Liu Y., Wu Y. (2025). Gamma-Aminobutyric Acid Attenuates Cortisol-Induced Damage in Human Colorectal Adenocarcinoma Cells via Nrf2 Signaling. Sci. Nat..

[58] Fridovich I. (1997). Superoxide Anion Radical (O_2_^−^), Superoxide Dismutases, and Related Matters. J. Biol. Chem..

[59] Jung D.J.S., Kim D.H., Beak S.H., Cho I.G., Hong S.J., Lee J., Lee J.O., Kim H.J., Malekkhahi M., Baik M. (2023). Effects of Vitamin E and Selenium Administration on Transportation Stress in Pregnant Dairy Heifers. J. Dairy Sci..

[60] Purwar V., Oberoi P.S., Dang A.K. (2017). Effect of Feed Supplement and Additives on Stress Mitigation in Karan Fries Heifers. Vet. World..

